# Anti-Obesity Effects of Metformin: A Scoping Review Evaluating the Feasibility of Brown Adipose Tissue as a Therapeutic Target

**DOI:** 10.3390/ijms24032227

**Published:** 2023-01-23

**Authors:** Khanyisani Ziqubu, Sithandiwe E. Mazibuko-Mbeje, Sinenhlanhla X. H. Mthembu, Sihle E. Mabhida, Babalwa U. Jack, Tawanda M. Nyambuya, Bongani B. Nkambule, Albertus K. Basson, Luca Tiano, Phiwayinkosi V. Dludla

**Affiliations:** 1 Biomedical Research and Innovation Platform, South African Medical Research Council, Tygerberg 7505, South Africa; 2 Department of Biochemistry, North-West University, Mmabatho 2745, South Africa; 3 Department of Life and Environmental Sciences, Polytechnic University of Marche, 60131 Ancona, Italy; 4 Department of Health Sciences, Namibia University of Science and Technology, Windhoek 9000, Namibia; 5 School of Laboratory Medicine and Medical Sciences, University of KwaZulu-Natal, Durban 4000, South Africa; 6 Department of Biochemistry and Microbiology, University of Zululand, KwaDlangezwa 3880, South Africa

**Keywords:** metformin, obesity, thermogenesis, brown adipose tissue, metabolism, therapeutic target

## Abstract

Brown adipose tissue (BAT) is increasingly recognized as the major therapeutic target to promote energy expenditure and ameliorate diverse metabolic complications. There is a general interest in understanding the pleiotropic effects of metformin against metabolic complications. Major electronic databases and search engines such as PubMed/MEDLINE, Google Scholar, and the Cochrane library were used to retrieve and critically discuss evidence reporting on the impact of metformin on regulating BAT thermogenic activity to ameliorate complications linked with obesity. The summarized evidence suggests that metformin can reduce body weight, enhance insulin sensitivity, and improve glucose metabolism by promoting BAT thermogenic activity in preclinical models of obesity. Notably, this anti-diabetic agent can affect the expression of major thermogenic transcriptional factors such as uncoupling protein 1 (UCP1), nuclear respiratory factor 1 (NRF1), and peroxisome-proliferator-activated receptor gamma coactivator 1-alpha (PGC1-α) to improve BAT mitochondrial function and promote energy expenditure. Interestingly, vital molecular markers involved in glucose metabolism and energy regulation such as AMP-activated protein kinase (AMPK) and fibroblast growth factor 21 (FGF21) are similarly upregulated by metformin treatment in preclinical models of obesity. The current review also discusses the clinical relevance of BAT and thermogenesis as therapeutic targets. This review explored critical components including effective dosage and appropriate intervention period, consistent with the beneficial effects of metformin against obesity-associated complications.

## 1. Introduction

Obesity persists as a serious global public health issue associated with the development of adverse health outcomes [1]. Based on the body mass index (BMI) ≥ 30 kg/m^2^ as a measure of obesity in humans, the World Health Organisation (WHO) reported that over 1.9 billion adults, approximately 39% of the adult population, were overweight by the year 2016 [2]. Obesity is broadly defined as a condition in which the intake of energy surpasses its use, a process that normally drives excessive body fat accumulation, leading to impaired metabolic function [3,4]. Arising metabolic anomalies are consistent with the development of metabolic diseases, including insulin resistance, non-alcoholic fatty liver diseases, type 2 diabetes (T2D), and cardiovascular diseases (CVDs) [3,4]. Evidently, adipose tissue has been subject to increasing research to explore the pathological features of obesity and metabolic syndrome [5,6]. As a result, comparative analysis of adipose tissue compartments or its distribution within the body, including the effect of therapeutic drugs on adipose tissue function, have become equally important [5,6,7].

Briefly, the two classical types of adipose tissue that have been characterized in the human body are white adipose tissue and brown adipose tissue (BAT) [8]. In terms of morphology and function, the former contains less mitochondria and unilocular lipid droplet to store excess energy as fat, whereas BAT has multilocular lipid droplets and is rich in mitochondria, which are equipped with uncoupling protein (UCP)-1 to uncouple oxidative phosphorylation from ATP synthesis, thereby dissipating energy as heat via a process known as thermogenesis [8]. In fact, physical activity has been linked with physiological benefits against metabolic complications by targeting the adipose tissue, thus promoting energy expenditure, as well as improving mitochondrial function, in part by prompting a phenotypic switch from energy-storing white adipose tissue (WAT) to thermogenic BAT [9,10,11]. Anyhow, the prevailing hypothesis suggests that promoting the thermogenic activity of BAT or WAT browning is consistent with enhanced energy expenditure, and this process could reverse some pathological features of metabolic syndrome [12,13,14]. Therefore, BAT has become an attractive target tissue to study obesity and associated metabolic complications [15,16].

Metformin, a biguanide derivate, remains as the most-prescribed anti-diabetic drugs, which is generally considered as a first-line pharmacotherapy in the treatment of T2D, in particular for individuals who are overweight and obese [17]. Beyond its well-known insulin-sensitizing and blood-glucose-lowering effects in in vitro and in vivo models of T2D, metformin takes pleiotropic actions and exerts multiple health benefits against obesity, cancers, liver diseases, cardiovascular disease, and renal diseases; this has been reviewed elsewhere [18,19]. In many trials, metformin has been shown to reduce weight gain and promote weight loss in obese subjects with or without T2D [20,21,22,23]. Accordingly, several well-elaborated systematic reviews have provided an insight into the use of metformin for weight management and the treatment of obesity [24,25,26,27]. However, the mechanisms of the drug’s action are not completely understood. Metformin could exert its anti-obesity effect through targeting and modulating adipose tissue function [28,29]. In obese mice, metformin decreased body weight and improved the metabolic profile by suppressing white adipocyte differentiation by affecting fibroblast growth factor 21 (FGF21), a key metabolic hormone that improves lipolysis in WAT and prevents fat accumulation [30]. Subsequently, several well-designed studies have shown that metformin may prevent weight gain in preclinical models of obesity by increasing the metabolic activity of BAT in a manner that is dependent and independent of the distinct action of UCP1, which is a molecular marker able to dissipate chemically bound energy as heat [31,32,33,34]. In line with establishing whether BAT is a plausible target for the treatment of obesity in humans, the current scoping review was conducted to further decipher and elaborate on the potential therapeutic mechanisms linked with the anti-obesity effects of metformin.

## 2. Methods: Search Strategy, Study Eligibility Criteria, and Data Items

**Figure 1:**
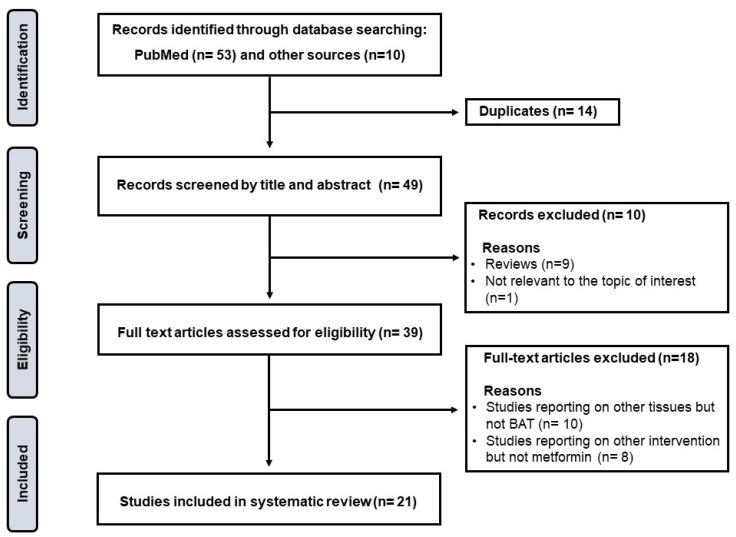
Schematic representation of the study’s selection procedure. In summary, after removing duplicates, only 49 studies were screened; of these, 39 full-text articles were assessed for eligibility, and only 21 articles met the inclusion criteria and are critically discussed with the current review.

The Preferred Reporting Items for Systematic reviews and Meta-Analyses (PRISMA) guidelines were followed to conduct this scoping review, and these are found at https://www.prisma-statement.org/Extensions/ScopingReviews?AspxAutoDetectCookieSupport=1 (Supplementary Materials, accessed on 10 January 2023). The protocol for the current review was not registered; however, well-established online databases such as The International Prospective Register of Systematic Reviews (PROSPERO) were accessed and screened to identify or eliminate any similar reviews being conducted. Briefly, a systematic search was conducted by two independent reviewers, in consultation with an experienced librarian, through major electronic databases and search engines such as PubMed/MEDLINE, Google Scholar, and the Cochrane library to retrieve relevant studies on the impact of metformin on regulating BAT thermogenic activity to modulate complications linked with obesity. The following search terms, including relevant synonyms, were applied: “metformin”, “brown adipose tissue”, and “obesity”. Studies reporting on other tissues other than BAT were excluded, as demonstrate in Figure 1. To enhance the relevance of the current manuscript, review papers were only screened for primary studies, whilst letters to the editor were excluded. In this review, particular attention was given to the experimental evidence highlighting the role of metformin in targeting BAT to combat obesity and its sequelae. Thus, extracted data items (from relevant literature) include the experimental model used, the dose and intervention period for metformin, as well as the main findings for the intervention concerning its potential antiobesity effects.

## 3. The Significance of BAT and Thermogenesis as a Therapeutic Target

**Figure 2:**
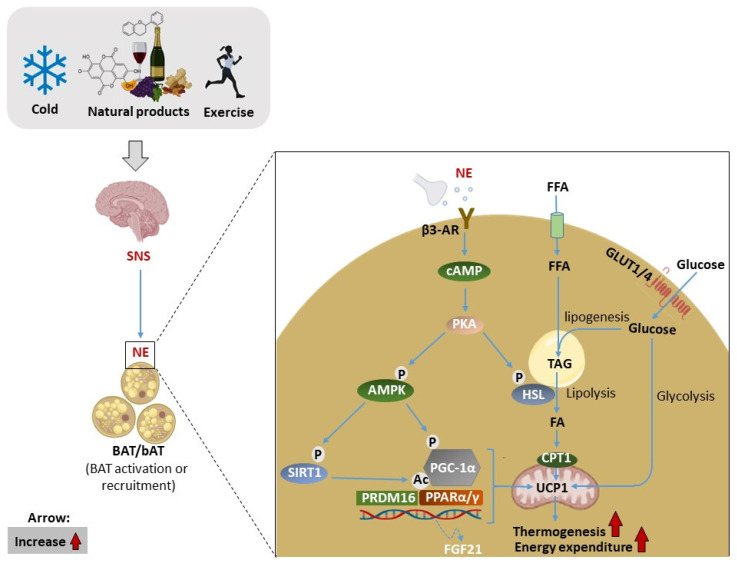
Illustration of the general mechanism of brown adipose tissue (BAT) activation and recruitment, as well as the other molecular mechanisms involved. Briefly, cold exposure, exercise, natural/pharmacological products, and other stimuli can activate sympathetic neurons innervating BAT release norepinephrine (NE), which binds to β3-adrenergic receptors (β3-AR) converting ATP to cyclic adenosine monophosphate (cAMP). Subsequently, cAMP activates protein kinase A (PKA), which then activate hormone-sensitive lipase (HSL) to liberate fatty acids (FAs) from triacylglyceride (TAG) stores through lipolysis, which in turn upregulate uncoupling protein 1 (UCP1) located in the mitochondria. Subsequently, the uptake of circulating free fatty acids (FFAs) and glucose contributes to the regeneration of intracellular triglyceride stores. Glucose is transported into the cell by glucose transporters (GLUTs), while FFAs are transported via cluster of differentiation 36 (CD36). On the other hand, activation of AM-activated protein kinase (AMPK) induces the complex of adipogenic and thermogenic transcriptional factors such as NAD-dependent deacetylase sirtuin-1 (SIRT1), proliferator-activated receptor gamma coactivator 1-alpha (PGC1α), and transcriptional factor PR domain containing 16 (PRDM16), which in turn increase UCP-1-driven thermogenesis and energy expenditure.

Brown adipose tissue (BAT), of which its primary function is to promote thermogenesis upon cold exposure, is a distinct type of adipose tissue that is widely viewed as a promising therapeutic target for obesity [35,36]. Initially, BAT was merely known to exist in hibernating marmots, where it was described as “neither fat nor flesh” by Swiss researchers [37]. More information was accumulated owing to the rediscovery of functionally active BAT in adult humans by virtue of technological advancements in clinical research using whole-body positron emission tomography [38,39,40]. As a result, harnessing the capacity of BAT to consume energy via WAT browning or BAT activation has been proposed as an ideal strategy to combat obesity [35,41,42,43]. The plausibility of this approach is driven by the unique capacity of BAT to increase energy expenditure and burn excess fat via a process referred to as “thermogenesis”. Figure 2 highlights some of the molecular mechanisms proposed to be involved in BAT activation, in part through the interactions of various targets/subunits such as cyclic adenosine monophosphate-protein kinase A (cAMP-PKA), peroxisome-proliferator-activated receptors (PPARs), PR domain containing 16 (PRDM16), peroxisome-proliferator-activated receptor gamma coactivator 1-alpha (PGC1α), as well as UCP1 [44,45]. Among the variety of metabolic substrates that are utilized by BAT, glucose and fatty acids are considered important fuel sources [46]. In fact, optimal uptake and utilization of these substrates can improve metabolic health [47,48]. For example, increased glucose uptake and non-esterified fatty acid turnover during acute cold exposure in humans or in experimental models of obesity are consistent with BAT activation [49,50]. Mechanically, adrenergic stimulation of β3-AR can induce glucose uptake in brown adipocytes via a glucose transporter (GLUT)-1 and GLUT4 [51]. On the other hand, a study evaluating FA uptake in human supraclavicular BAT during cold exposure demonstrated that basal and cold-induced FA uptake is impaired in obese subjects [52]. This could be explained by the whitening and impaired recruitment of BAT in obesity [53,54]. In addition to sympathetic activation of the β3-AR and cAMP/PKA signaling pathway, the 5’ adenosine monophosphate-activated protein kinase (AMPK) pathway is a downstream signaling pathway that also plays an important role in regulating substrate utilization and energy metabolism [44]. This mechanism has been studied under the context of WAT browning [55].

Over the years, more research has been performed to identify the specific potent pharmacological products that mimic the cold effects on BAT with minimal or no side effects. For example, the semi-chronic treatment with selective β3-AR agonist CL316243 in rodents did not achieve local transformation of WAT to a BAT-like phenotype without systemic exposure. However, it remains to be established whether this observation was also relevant for other β3-AR agonists and other species, especially humans [56]. To develop 3-AR agonists for application in humans, mirabegron, a β3-AR agonist, was approved by the United States Food and Drug Administration to treat overactive bladder and was found to increase BAT glucose uptake and energy expenditure in healthy humans [57,58]. However, this was accompanied by the adverse effect of increased blood pressure. Alternatively, several plant-derived products, in particularly polyphenols, can influence BAT function and recruitment via the AMPK pathway [59,60]. As reviewed by Zhang et al. [42], flavonoids can promote BAT thermogenesis and induce WAT browning via the AMPK-PGC-1α/Sirt1 and PPAR-α/γ signaling pathways upon sympathetic nervous system activation, which endorses the release of adrenaline and thyroid hormones. Moreover, resveratrol from the class of polyphenolic compounds called stilbenes can directly activate AMPK to induce beige adipocytes formation in WAT, resulting in enhanced glucose uptake and energy consumption in mice [61]. Alternatively, physical exercise is another highly recommended intervention strategy to mitigate and eradicate metabolic disease [10,11,62]. Acute exercise has been shown to increase circulating FGF21 in both mice and humans [63], a cold-induced endocrine activators of BAT function, which is mainly secreted in the liver, but also expressed and released in BAT during thermogenic activation [64]. FGF21 integrates several metabolic pathways allowing the regulation of glucose levels and lipid metabolism, as well as whole-body energy homeostasis [65,66]. A study by Liu et al. [67] demonstrated that pharmacological treatment with FGF21 strongly improves plasma cholesterol metabolism to reduce atherosclerosis via BAT activation and WAT browning. Recently, the therapeutic potential of FGF21 has been evaluated with the development of recombinant FGF21 analogs. Indeed, Kaufman et al. [68] reported that AKR-001, an Fc-FGF21 analogue, exerts a sustained pharmacodynamic effect on insulin sensitivity and lipid metabolism in patients with T2D. However, the clinical profile of AKR-001 requires further evaluation as a therapeutic intervention for metabolic diseases.

## 4. An Overview of Metformin and Its Therapeutic Potential against Metabolic Diseases

**Figure 3:**
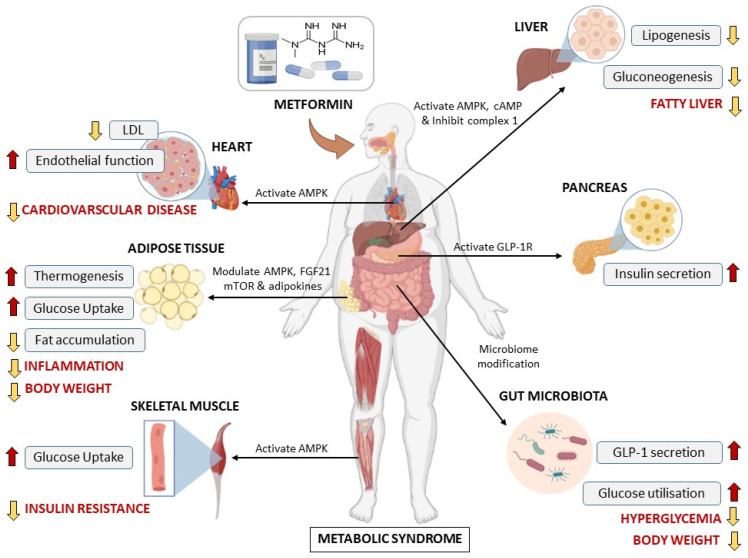
An overview of the most prominent mechanisms of action and impact of metformin on different metabolic diseases in conditions of metabolic syndrome. AMPK: AMP-activated protein kinase, cAMP: cyclic adenosine monophosphate, FGF21: fibroblast growth factor 21, GLP-1R: glucagon-like peptide 1 receptor, LDL: low-density lipoprotein.

Metformin also known as 1,1-dimethylbiguanide hydrochloride is a widely prescribed oral anti-diabetic drug, which was approved by the U.S. Food Drug Administration to be used by adults and children aged >10 years [69]. Historically, metformin was first discovered in the 1920s, its history was linked to the traditional herbal medicine found in Europe known as *Galega officinalis* [69]. This herbal medicine was popular in the 1918s because of its blood-glucose-lowering properties, and it was found to be rich in guanidine [69,70]. Some of the guanidine derivatives such as metformin were synthesized and used to treat diabetes due to their glucose-lowering benefit in the 1920s and 1930s [69,71]. Among these derivatives, metformin was considered weak, and its use was limited compared to the other biguanides. For this reason, metformin was then forgotten for years, until the time when other biguanides (phenformin and buformin) were withdrawn from the market in the late 1970s due to their association with lactic acidosis, although they had more potent activities than metformin [72,73]. Around the 1980s and early 1990s, an accumulative number of studies demonstrated the efficient antihyperglycemic effects of metformin without overt side effects including hypoglycemia and weight gain, and this resulted in the rescue of metformin and support for its clinical use [69,70,72,73]. After these ground-breaking studies, metformin has remained the most widely prescribed drug for T2D with an excellent safety and tolerability profile, and it has since been added to the World Health Organization’s list of essential medicines in 2011 [74]. Over the years, metformin has gained more recognition, not only as a blood-glucose-lowering agent, but because of its pleiotropic effects in modulating diverse metabolic complications ranging from obesity, insulin resistance, myocardial complications, liver steatosis, to polycystic ovary syndrome [18,75,76]. Although the mechanisms underlying these health benefits are complex and not completely understood, some mechanisms by which metformin alleviates various metabolic disease have been proposed (Figure 3). These include activation of AMPK, inhibition of the complex I mitochondrial transport chain, and antagonizing glucagon-induced cAMP, which lead to the amelioration of glycemic control. as extensively reviewed elsewhere [75,77,78,79,80].

According to Agius et al., 2020 [81], the AMPK signaling pathway is one of the most extensively studied classical mechanism of metformin. Basically, AMPK is a central regulator of energy homeostasis, and it is recognized as a major regulator of lipid biosynthetic pathways due to its role in the phosphorylation and inactivation of key enzymes such as acetyl-CoA carboxylase that play a pivotal role in the regulation of fatty acid metabolism [82,83]. Several studies have proposed that the activation of AMPK by metformin could be associated with its accumulation in the mitochondria because of its positive charge at physiological pH, causing the effective modulation of the respiratory chain, a process that, in turn, regulates a range of other related target proteins [81,82]. Although the metformin-induced activation of AMPK is a well-documented mechanism [83], it may not account for all the actions of the drug. There are many other AMPK-independent mechanisms underlying the action of metformin [19,83]. For example, beyond its increasingly reported cardioprotective effects [84,85,86], metformin displayed anti-obesity effects in multipotent C3H10T1/2 MSC by exerting reciprocal control over the activities of osteogenic transcription factor Runt-related transcription factor 2 and the adipogenic transcription factor PPARγ, leading to the suppression of adipogenesis [87]. These effects appeared to be independent of AMPK activation, but rather through the suppression of the mammalian target of rapamycin (mTOR)/p70S6K (a mitogen-activated Ser/Thr protein kinase) signaling pathway [88]. The anti-obesity effects of metformin have garnered more interest and have been tested in human subjects [89,90], and some of the underlying mechanisms were reviewed by Yerevanian and colleagues [27]. Accordingly, it has been strongly hypothesized and confirmed that metformin could exert an anti-obesity effect via gut microbiome modulation in various studies both in diabetic and non-diabetic human subjects and animals [22,91,92]. The amelioration of metabolic syndrome by metformin is associated with reduced indices of low-grade inflammation independent of the gut microbiota [76]. Further exploration of the mechanisms underlying the anti-obesity or weight-loss-inducing effects of metformin is necessary to identify new pharmacologic targets for obesity and its sequelae. In recent years, some research supported that BAT may be a target of metformin. Indeed, emerging experimental evidence suggests that metformin can reduce body weight and enhance energy expenditure via the activation of BAT or browning of WAT [29,31,93].

## 5. Results: Impact of Metformin on Energy Expenditure and BAT Activity

### 5.1. General Characteristics and Overview of Included Literature

Currently, diverse preclinical models are applied to investigate the pathophysiological mechanisms of the disease or the therapeutic effects of drugs against obesity [94,95,96]. As such, progressive analysis of the literature has been conducted to update the strengths and limitations of some commonly explored experimental models of obesity. Indeed, from the initial evidence that was published from the 1960s looking at body composition as a model and estimation for obesity [97], there are now approximately “34,330” results that can be retrieved through a PubMed search, under the heading “experimental models of obesity” [94]. Notably, diet-induced obesity in animals or rodents appears to be the predominant system to explore the pathological features of obesity, as reviewed elsewhere [98,99]. Exposing mice or rats to an obesogenic diet has been associated with increased body weight, which predominantly characterizes excessive fat accumulation or ectopic lipid accumulation, which, depending on the composition of the diet or the time of exposure to this diet, may occur together with other metabolic complications such as hyperglycemia, dyslipidemia, and hormonal dysregulations [95,98]. Besides experimental models of diet-induced obesity, gene-specific mutations have been another approach that has been explored to uncover the therapeutic effects of drugs against T2D or metabolic syndrome. Certainly, Zucker rats, as well as obese (*ob*/*ob*) and diabetic (*db*/*db*) mice have been progressively used in experiments based on their characteristic features of spontaneously developing obesity or metabolic complications such as hyperphagia, insulin resistance, impaired glucose tolerance, and cardiovascular complications. The result section details the aforementioned preclinical models, to explore the anti-obesity properties of metformin and its capacity to regulate energy expenditure, in part by targeting the brown adipose tissue. In terms of quality of evidence, all included preclinical studies could be trusted based on their design and applied statistical analysis, which were appropriate.

### 5.2. Evidence on the Short-Term Treatment Effects of Metformin

From the systematic search of the literature, evidence on the therapeutic effects of metformin emerged as early as 1993 [100], showing that short-term (duration equivalent to 2 weeks or less) treatment with metformin (320 mg/kg/day for 12 days) could reduce body weight and cumulative food intake in obese Zucker rats. Although these effects were positive, this study showed that metformin did not affect thermogenesis measured using the binding of [^3^H] GDP to BAT mitochondria or the mRNA expression of UCPs within BAT. Although evidence suggests that metformin could reduce food intake in another genetic model of obesity (*ob*/*ob* mice), Kumar and colleagues [101] showed that metformin (200 mg/kg/day for 10 days) did not affect serum glucose levels or the expression of nitric oxide synthase in the BAT of obese mice. These findings showed that metformin can partially affect body weight or reduce food intake; however, there are apparent limitations in its therapeutic impact on regulating energy expenditure, or specifically the thermogenic activity of BAT. The major limitation could be related to the very short treatment time (≤12 days) or the fact that only a few parameters were used to measure the thermogenic activity of BAT.

Nonetheless, follow-up studies by other groups on the short-term effects of metformin showed positive results, especially on the modulation of the thermogenic activity of BAT. For example, using SV40T-immortalized brown adipocytes from the FVB strain of mice, Klein and colleagues [102] showed that directly treating these cells with metformin (500 µM and 1 mM for 8 days) could dose dependently block leptin secretion while acutely stimulating mitogen-activated protein kinases (p44/p42 MAPK), the major pathway involved in the survival signals that counteract cell death [103]. Furthermore, Hu and colleagues [104] showed that, apart from preventing weight gain or the loss of BAT, metformin (300 mg/kg for 2 weeks) could upregulate BAT genes involved in energy expenditure such as AMPK and UCP3 and those cited in the regulation of lipid metabolism such as resistin, fatty acid synthase, insulin-induced gene 2, CCAAT/enhancer binding protein alpha (C/EBPa), and PPARγ in Sprague Dawley rats. Such findings were confirmed by others showing that metformin is possibly taken up by BAT cells [31], and its short-term treatment (duration equivalent to 2 weeks or less) is consistent with the enhanced cellular oxygen capacity of BAT or the elevated expression of its thermogenic transcriptional factors such as Prdm16 and UCP1 in preclinical models of obesity [32,105], further implying that the anti-obesity properties of metformin are likely modulated through its capacity to enhance BAT thermogenic activity.

### 5.3. Evidence on the Long-Term Treatment Effects of Metformin

Generate evidence gives an overview of preclinical studies on the long-term effects of metformin, especially its capacity to affect obesity-related complications by regulating energy expenditure and BAT. In fact, with evidence opposing the findings on the short-term effects of metformin on Zucker rats [100], Savontaus and co-workers [106] showed that this anti-diabetic drug (at 300 mg/kg/day for 3 weeks) could reduce body weight and cumulative food intake in a similar model of obese Zucker rats. Thereafter, work from other groups [33,107], using mice treated with metformin (at 200 mg/kg body weight/day for 4 weeks), showed that this anti-diabetic drug could improve lipid profiles by reducing plasma total cholesterol and triglyceride levels, while decreasing BAT mass and lipid droplets. These positive effects were concomitant to enhanced BAT thermogenic activity, which was modulated in part through the upregulation of UCP1 expression or AMPK activation. Besides being major players in the regulation of energy expenditure through interacting with other pathways [108,109], both UCP1 and AMPK are crucial in metabolism and are increasingly recognized as therapeutic targets in protecting against metabolic complications [110,111].

The presented evidence further elucidates other diverse therapeutic mechanisms by which metformin can affect metabolic activity, in addition to promoting BAT thermogenic activity in various preclinical models of obesity. For instance, studies [29,30,112,113] making use of obese mice treated with metformin (at 50–250 mg/kg/day) for at least 8 weeks showed that this anti-diabetic agent could improve glucose metabolism and insulin sensitivity or hinder white adipocyte differentiation via the induction of fibroblast growth factor (FGF) 21 or enhancing mRNA expression of perilipin 5 in BAT. On the other hand, Karise and co-workers [34] showed that metformin treatment (at 250 mg/kg/day for 8 weeks) further stimulated FGF21 to enhance AMPK activity and improved mitochondrial biogenesis within BAT, in part by upregulating the expression of nuclear respiratory factor (NRF)1, transcription factor A, mitochondrial (TFAM), UCP1, and PGC1-α in obese mice. While the significant role of transcriptional factors such as NRF1, UCP1, and PGC1-α play an important role in promoting the thermogenic activity of BAT [114,115], available evidence indicates that the therapeutic effects of metformin may extend to improving glucose metabolism through the modulation of other factors such as FGF21 and perilipin in preclinical models [116,117]. Overall, the evidence affirms that the therapeutic effects of metformin (at 100–200 mg/kg/day) treatment for at least 4 weeks could improve glucose homeostasis and the lipid profiles, while also reducing inflammatory features in BAT such as macrophage infiltration, proinflammatory signaling and gene expression, and increasing systemic energy expenditure and BAT activation in various preclinical models of obesity [118,119,120].

## 6. Clinical Translation of Results

Preclinical models of obesity have become relevant to uncover the efficacy of diverse drugs or bioactive compounds against metabolic diseases, especially when directly exploring the implicated molecular mechanisms in response to drug treatment [94,95,96]. However, the generated data must still be confirmed in well-organized clinical trials to better understand and confirm the therapeutic properties of these agents. In fact, organizations such as the U.S. Food and Drug Administration, which are primarily responsible for approving the use of most therapeutic drugs in human subjects, have a set of guidelines to follow before any therapeutic drug can be approved [121]. In addition to testing new drugs on preclinical models, clinical trials provide an essential scientific foundation for the further development of any therapeutic drug. Although metformin was long approved as a remedy for diabetes [122], there is still a general interest in understanding its therapeutic mechanisms, especially its acknowledged pleiotropic effects [19]. While it is acknowledged that metformin can promote weight loss in obese patients or improve metabolic function in subjects with metabolic syndrome, the implicated therapeutic mechanisms still need to be resolved. Others have argued that these effects can be through effective modulation of adipokines, leading to enhanced energy expenditure and improved insulin sensitivity in obese patients with T2D [28,69,123,124]. There is currently limited evidence on the implications of BAT regulation in subjects with obesity or patients with metabolic syndrome treated with metformin. Two clinical studies have been reported, with Srinivasa and colleagues [125] showing that metformin (at 500–850 mg twice daily for 12 months) could improve energy homoeostasis, in part by enhancing the expression of UCP1 and circulating levels of FGF21 dorsocervical subcutaneous fat biopsies in HIV-infected patients presenting metabolic complications. However, Oliveira and co-workers [93] showed that short-term treatment with this anti-diabetic drug (at 1500 mg/day for 60 days) could not affect BAT activity and plasma irisin levels in women with polycystic ovary syndrome. Consistent with some preclinical evidence [100,101], it was further suggested that long-term treatment with metformin is more effective than short-term treatment. Table 1 gives an overview of preclinical evidence on the anti-obesity properties of metformin and its capacity to regulate energy expenditure through brown adipose tissue. Apparently, such information must be confirmed in well-designed clinical trials.

## 7. Conclusions and Future Perspective

**Figure 4:**
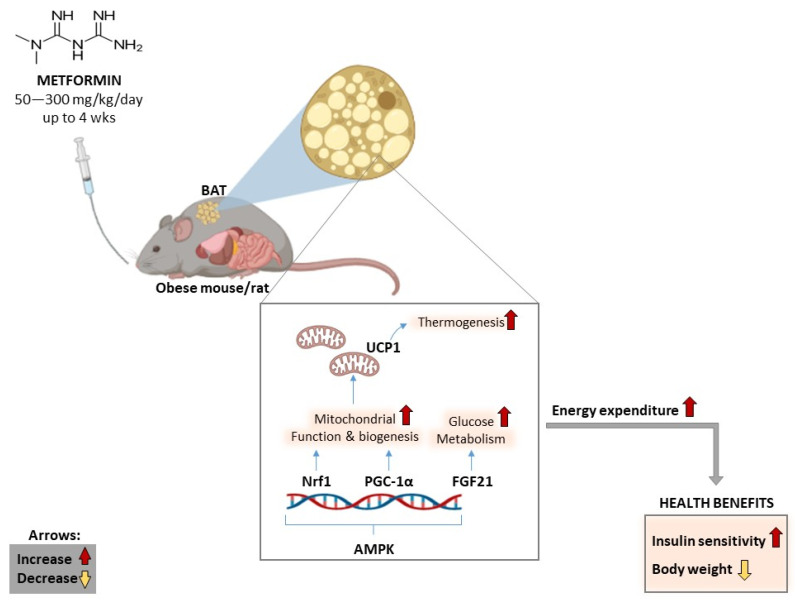
The main putative molecular mechanisms involved in the anti-obesity effects of metformin mediated by brown adipose tissue function. AMPK: AMP-activated protein kinase, FGF21: fibroblast growth factor 21, Nrf1: nuclear respiratory factor 1: PGC-1α, peroxisome-proliferator-activated receptor gamma coactivator 1-alpha, UCP1: uncoupling protein 1, mg: milligram, kg; kilogram, wks: weeks.

The evidence summarized in Table 1 affirms that metformin displays anti-obesity properties and has the capacity to reduce body weight and modulate glucose metabolism, in part by targeting BAT to improve mitochondrial function and promote energy expenditure in various preclinical models of obesity (Figure 4). In terms of molecular markers, activation or enhanced expression of AMPK and FGF21, together with effective regulation of thermogenic markers such as UCP1, NRF1, and PGC1α within BAT appear to be the predominant mechanisms by which metformin exerts its therapeutic effects. Notably, consistent results are achieved whether metformin is administered through oral gavage in drinking water or supplemented in diet, with effective doses ranging from 50–300 mg/kg/day (in both mice and rats), for an approximate period of 4 weeks. Preliminary data suggest that this biguanide class of drugs can improve energy homoeostasis, in part by enhancing the expression of thermogenic factors such as UCP1 and FGF21 in dorsocervical subcutaneous fat biopsies [125]. However, the summarized literature is not without limitations. Firstly, although preclinical studies provide an important platform to elucidate or understand the potential therapeutic mechanisms for any drug (including metformin), such information still needs to be confirmed in larger and well-organized clinical trials. Thus, additional studies are required to confirm these therapeutic effects. Information relevant to how metformin performs in comparison or in combination with other potential drug agents such as natural products that are known to be rich in antioxidants and anti-inflammatory activities is also of interest and remains to be explored.

## Figures and Tables

**Table 1:** An overview of preclinical studies on the anti-obesity properties of metformin and its capacity to regulate energy expenditure through brown adipose tissue.

Author, Year	Experimental Model and Metformin Dose	Main Findings
**Short-term effects of metformin (treatment duration equivalent to 2 weeks or less)**		
**Rouru et al., 1993 [100]**	Obese Zucker rats treated with metformin (dissolved in drinking water) at 320 mg/kg/day for 12 days	Reduced body weight and cumulative food intake; however, did not affect thermogenesis, measured using the binding of [^3^H]GDP to BAT mitochondria and the expression of uncoupling protein mRNA in brown adipose tissue (BAT).
**Kumar et al., 2001 [101]**	Genetically modified obese (*ob*/*ob*) mice treated with metformin (subcutaneously) at 200 mg/kg/day for 10 days	Reduced food intake, but did not affect serum glucose levels. Metformin did not affect the expression of nitric oxide synthase in the BAT of obese mice.
**Klein et al., 2004 [102]**	SV40T-immortalized brown adipocytes from the FVB strain of mice were treated with metformin (500 µM and 1 mM) for 8 days	Dose dependently reduced leptin secretion without affecting adipocyte differentiation. Metformin also acutely stimulated p44/p42 MAP kinase and inhibited leptin secretion in a dose-dependent manner in BAT.
**Hu et al., 2014 [104]**	Olanzapine-induced weigh gain in Sprague Dawley rats treated with metformin (oral gavage) at 300 mg/kg for 2 weeks	Prevented weight gain and loss of BAT. Mechanistically, metformin upregulated BAT genes involved in energy expenditure such as AMP-activated protein kinase (AMPK) and uncoupling protein (UCP)3 and those cited in the regulation of lipid metabolism such as resistin, fatty acid synthase, insulin-induced gene 2, CCAAT/enhancer binding protein alpha (C/EBPa), and peroxisome-proliferator-activated receptor gamma (PPARγ)
**Yang et al., 2016 [105]**	Newborn offspring of C57BL/6 mice fed a high-fat diet (HFD) and injected intraperitoneally with metformin at 250 mg/kg for 15 consecutive days	Rescued obesity-induced suppression of brown adipogenesis and thermogenesis. Metformin also activated AMPKα and upregulated the expression of PR domain containing 16 (Prdm16) in BAT.
**Tokubuchi et al., 2017 [32]**	Sprague Dawley rats were treated with metformin (dissolved in drinking water) at 2.5 mg/mL for 2 weeks	Increased plasma levels of lactate and pyruvate. Metformin also significantly reduced visceral fat mass, upregulated fat oxidation-related enzyme in the liver, UCP1 in BAT, and UCP3 in the skeletal muscle.
**Breining et al., 2018 [31]**	Organic cation transporter (Oct)1/2^−/−^ mice on an FVB background received [^11^C]-metformin (0.2–1.0 GBq) containing 0.1–0.5 µg/mL metformin for 60 min, whereas, brown adipocytes of human origin were treated with metformin (0, 0.1 or 0.5 mM) for 24 h	Metformin was taken up in murine interscapular BAT depots, and this was associated with increased expression of UCP1. Notably, metformin reduced cellular oxygen consumption in human brown adipocyte cells.
**Long-term effects of metformin (treatment duration equivalent to >2 weeks)**		
**Savontaus et al., 1998 [106]**	Obese Zucker rats treated with metformin (dissolved in drinking water) at 300 mg/kg/day for 3 weeks	Reduced weight gain, as well as food and water intake; however, did not affect mRNA expressions of UCP1, UCP2, or UCP3 in BAT. The observed effect of metformin on the expression of UCPs was when combined with β3-adrenoceptor agonist (BRL 35135) when administered at 0.5 mg/kg/day.
**Geerling et al., 2014 [107]**	E3L. CETP mice fed a Western-type diet supplemented with 200 mg/kg body weight/day (0.2%, *w*/*w*) metformin for 4 weeks	Lowered plasma total cholesterol and triglyceride levels, in addition to reducing BAT mass and lipid droplet. This effect was linked to increases in AMP-activated protein kinase a1 (AMPKa1) expression and activity, including hormone-sensitive lipase and mitochondrial respiratory chain complexes in BAT.
**Liang et al., 2016 [33]**	Offspring of C57/BL mice fed an HFD and treated with metformin (dissolved in saline) at 200 mg/kg for 21 days	Decreased serotonin concentration and promoted BAT thermogenic activity by upregulating the expression of UCP1.
**Kim et al., 2016 [30]**	C57BL/6 mice fed an HFD and treated with metformin (oral gavage) at 10 mg/kg or 50 mg/kg for 14 weeks	Improved glucose metabolism and suppressed white adipocyte differentiation via induction of fibroblast growth factor (FGF) 21 in the liver and in white adipocytes.
**Mehdi et al., 2018 [112]**	C57BL/6 J mice treated with metformin (oral gavage) at 250 mg/kg/day for 45 days	Significantly increased the mRNA expression of perilipin 5 in BAT.
**Kim et al., 2018 [113]**	Collagen-induced arthritis DBA/1J mice treated with metformin (oral gavage) at 50 mg/kg for 13 weeks	Displayed a small normalizing effect on the metabolic profile of obese mice. In addition, metformin promoted BAT differentiation while increasing the production of pAMPKα and fibroblast growth factor 21 (FGF21).
**Karise et al., 2019 [34]**	C57Bl/6 mice fed a HFD and treated with metformin (oral gavage) at 250 mg/kg/day for 8 weeks	Increased BAT content and function, as shown by an increase in adipocyte proliferation and differentiation. Metformin further promoted the activation of AMPK and enhanced thermogenic markers (UCP1 and PGC1-α) through adrenergic stimuli and FGF21. Metformin also improved mitochondrial biogenesis in BAT by upregulating nuclear respiratory factor (NRF) 1 and transcription factor A, mitochondrial (TFAM).
**Yuan et al., 2019 [29]**	C57BL/6J mice fed and treated with metformin (oral gavage) at 200 mg/kg/day for 8 weeks	Improved the body weight and insulin sensitivity, while affecting differential expression of 3486 proteins in BAT that were mainly assigned to the pathways of EIF2 signaling and mitochondrial dysfunction. Furthermore, carnitine palmitoyltransferase (CPT)1b and CPT2 in BAT were downregulated by metformin significantly.
**Abdel-Rehim et al., 2019 [118]**	Sprague Dawley rats fed an HFD and treated with metformin (oral gavage) at 200 mg/kg/day for 4 weeks	Improved glucose homeostasis and lipid profile parameters. Metformin also significantly reduced the expression of SREBP-1c, which regulates lipid synthesis in BAT.
**Stojnic et al., 2021 [119]**	C57BL/6J mice fed an HFD and metformin (dissolved in drinking water) at 100 mg/kg/day for 4 weeks	Improved glucose control and insulin sensitivity. Treatment did not affect energy intake, but increased systemic energy expenditure and BAT activation
**Pescador et al., 2021 [120]**	C75BL/6J mice fed HFD and treated with metformin (oral gavage) at 100 mg/kg/day for 6 weeks	Reduced inflammatory features in BAT such as macrophage infiltration, proinflammatory signaling, and gene expression and restored the response to cold exposure. Furthermore, suppressed a HIF1α-dependent pro-inflammatory program that was likely responsible for a secondary beneficial effect on insulin-mediated glucose uptake and β-adrenergic responses in BAT.

## Data Availability

Not applicable.

## Supplementary Materials

The following supporting information can be downloaded at: https://www.mdpi.com/article/10.3390/ijms24032227/s1. Reference [126] are cited in the Supplementary Materials.

## Abbreviations

AMP-activated protein kinase (AMPK), beta 3-adrenergic receptors (β3-AR), brown adipose tissue (BAT), cardiovascular disease (CVD), cyclic adenosine monophosphate (cAMP), free fatty acid (FFA), fibroblast growth factor 21 (FGF21), glucose transporter (GLUT), low-density lipoprotein (LDL), norepinephrine (NE), nuclear respiratory factor (NRF1), peroxisome proliferator activated receptor (PPAR), peroxisome-proliferator-activated receptor gamma coactivator 1-alpha (PGC1-α), protein kinase A (PKA), NAD-dependent deacetylase sirtuin-1 (Sirtuin1), type 2 diabetes (T2D), triacylglycerol (TAG), uncoupling protein 1 (UCP1), white adipose tissue (WAT).
